# Single Cell RNA Sequencing Reveals *THBS1*+*CD14*+ Monocyte Modulates Inflammatory Activation via NRLP3‐Inflammasome in Congenital Heart Block

**DOI:** 10.1111/jcmm.71270

**Published:** 2026-07-21

**Authors:** Sha Lin, Jie Tang, Chuan Wang, Yimin Hua, Haiyan Yu, Kaiyu Zhou, Yifei Li

**Affiliations:** ^1^ Department of Pediatrics, Ministry of Education Key Laboratory of Women and Children's Diseases and Birth Defects, West China Second University Hospital Sichuan University Chengdu Sichuan China; ^2^ Key Laboratory of Birth Defects and Related Diseases of Women and Children of MOE, Department of Obstetrics and Gynecology, West China Second University Hospital Sichuan University Chengdu Sichuan China

**Keywords:** congenital heart block, IL‐1β, NRLP3‐inflammasome, single cell RNA sequence, THBS1

## Abstract

Isolated congenital heart block (iCHB) is defined as atrioventricular block without structural cardiac defects, characterized by irreversible fibrosis of the cardiac conduction system. Maternal autoantibodies may elicit systemic exaggerated immune responses involving type I interferon (IFN) signalling cascade, yet peripheral circulating immunity in CHB pathogenesis remains poorly understood. To investigate this, we performed single‐cell RNA sequencing (scRNA‐seq), followed by differential expression gene (DEG) analysis, SCENIC analysis, pseudotime analysis and cell communication analysis, to characterize systemic immune alterations in foetuses with CHB treated with dexamethasone and validated key findings by real‐time quantitative PCR (qPCR) and flow cytometry. Compared with controls, CHB foetuses exhibited a markedly activated inflammatory response involving both IFN and NF‐κB pathways. Although monocytes showed significant changes in cellular proportions, upregulated DEGs and interferon‐stimulated genes, prioritized cellular responses and enhanced intercellular interactions. Notably, *THBS1+CD14+* monocytes had a pro‐inflammatory phenotype with upregulated NLRP3 inflammasome‐related genes and maturation toward a pro‐inflammatory state, and *Thbs1* conditional knockout mice showed reduced *IL‐1β* levels in BMDMs. Additionally, dexamethasone‐treated monocytes had downregulated *SOD2* (anti‐apoptotic) levels compared to controls, confirmed by qPCR, and flow cytometry verified that dexamethasone promoted monocyte apoptosis. In conclusion, the peripheral immune system in CHB is characterized by innate immune activation driven mainly by monocytes, along with systemic inflammation including IFN signalling. *THBS1+*/*CD14+* monocytes represent a distinct proinflammatory phenotype potentially linked to NLRP3‐mediated inflammatory responses.

## 1 Introduction

Isolated congenital heart block (iCHB) is defined as atrioventricular block (AVB) diagnosed during the fetal period or within 28 days after birth without pathogenic structural cardiac abnormalities, encompassing both idiopathic and immune‐mediated forms [1, 2]. The majority of iCHB cases are immune‐mediated, driven by maternal autoantibodies [3]. Among pregnant women positive for anti‐SSA/Ro autoantibodies, 1.7%–5% of foetuses develop AVB [4, 5, 6], most of which progress to complete atrioventricular block (CAVB) [4]. Once CAVB occurs, neonatal mortality can reach as high as 34% [7], and over 70% of affected children require pacemaker implantation before adulthood [4].

The pathogenesis of immune‐mediated CHB remains incompletely understood. The prevailing model proposes that maternal anti‐SSA/Ro and anti‐SSB/La autoantibodies cross the placenta, engage with fetal cardiac conduction cells, forming immune complexes that stimulate macrophage phagocytosis, triggering downstream inflammatory and fibrotic cascades that ultimately lead to chronic inflammation, fibrosis of the atrioventricular node, and irreversible progression of AVB [8]. However, the relatively low incidence of CHB even among autoantibody‐positive foetuses suggests the involvement of additional factors. Recent studies have begun to explore systemic immune‐inflammatory responses in CHB, including hypercytokinemia, activation of the type I interferon (IFN‐I) system, and activation of circulating innate immune cells such as natural killer (NK) cells and monocytes [9, 10]. Yet, a comprehensive understanding of how the fetal peripheral immune system responds—from the initial entry of maternal autoantibodies into the fetal circulation to the eventual development of cardiac inflammation and fibrosis—remains lacking. Moreover, idiopathic CHB also carries a substantial risk of progression to CAVB, and despite negative maternal autoantibody status, immunological perturbations may underlie many of these cases, albeit often manifesting as subtle alterations.

From a therapeutic standpoint, autoimmune CHB represents the only subtype of fetal AVB for which pharmacological intervention may improve prognosis and survival. Glucocorticoids such as dexamethasone have been suggested as a potential therapeutic approach upon diagnosis of autoimmune CHB [11]. But the unclear effect of dexamethasone in fetal and the variability of clinical outcome have led the therapeutic approach controversial. Given that CAVB can develop abruptly within 1 week following fetal echocardiography [12], the window for urgent prenatal intervention is extremely narrow. In our clinical practice, all foetuses diagnosed with CHB receive dexamethasone following diagnosis, irrespective of maternal autoantibody status, underscoring the urgent need to elucidate the cellular mechanisms underlying treatment response and disease pathogenesis.

Single‐cell transcriptomic technologies enable high‐resolution dissection of immune cell heterogeneity, facilitating the exploration of how inflammatory environments or cell‐intrinsic factors shape immune reactions in a cell‐specific manner under disease conditions. In this study, we performed single‐cell RNA sequencing (scRNA‐seq) on umbilical cord blood (UCB) from CHB patients who received dexamethasone during the fetal period, thereby filling the current gap in understanding the peripheral circulating immune landscape in iCHB, providing novel insights into its immunological basis and offering a valuable resource for future investigations.

## 2 Method

### 2.1 Ethics Approval and Consent to Participate

The study was reviewed and approved by the Ethics Committee of West China Second University Hospital with IRB Protocol Number 2021‐069.

### 2.2 Human Participant Inclusion Criteria and Blood Sample Processing

All donors were recruited from West China Second University Hospital between December 2022 and December 2023. The study was reviewed and approved by the Ethics Committee of West China Second University Hospital with IRB Protocol Number of 2021‐069. Informed consent was obtained from the donors and their guardians. Patients enrolled in this study were diagnosed with type II atrioventricular (AV) block under fetal echocardiography with no signs of structural abnormalities. The healthy umbilical cord blood samples single‐cell RNA sequencing datasets were downloaded from the Gene Expression Omnibus data repository under accession number *GSE157007*. UCB samples were obtained from three participants immediately after fetus delivery. The cord was single clamped near the placenta, and double clamped and cut near the infant. Approximately 10 mL each of UCB from each donor was collected in an EDTA‐anticoagulant tube and was transferred to the laboratory within 1 h with ice. To isolate UCB nucleated cells, Ficoll Paque Plus Buffer (20 mL; GE Inc.) was added to clean 50 mL tubes, and UCB samples diluted with an equal volume of phosphate‐buffered saline (PBS) were carefully added on top of each Ficoll buffer. After density gradient centrifugation, following which the supernatant was discarded, the cells were resuspended in 3 mL PBS. The cell viability should exceed 90% determined with trypan blue staining.

### 2.3 Single‐Cell RNA Sequencing and Analysis

The raw sequencing data were processed by the Cell Ranger pipeline (v6.1.2; 10× Genomics), including demultiplexing, genome alignment (GRCh38), barcode counting and unique molecular identifier (UMI) counting. The gene‐barcode matrix of UMI counts was then analysed with Seurat (v4.2.0) [13] for quality control, normalization, dimensional reduction, clustering and visualization. The batch effect was removed by package harmony (v1.0.0) [14], which is an algorithm that projects cells into a shared embedding in which cells group by cell type rather than dataset‐specific conditions. For all samples, the following criteria were applied for quality control to filter out low‐quality cells, erythrocyte and doublets: expressed less than 200 genes or greater than 4000 genes, erythrocyte gene percentage < 3%, and mitochondrial gene (MT‐) percentage < 10% (Figure S1). The count matrix was log‐normalized (*NormalizeData*), and the top 2000 most variable genes found by function *FindVariableGenes* were identified for dimensional reduction. The normalized data within each age group were then merged with the function *IntegrateData*, and finally merged into a master data object. Then the integrated matrix was scaled (*ScaleData*), following which the expression values of all genes in all cells were subjected to principal‐component analysis (PCA).

### 2.4 Clustering and Sub‐Clustering Analysis Using Seurat and UMAP Visualization

The PCA‐reduced data were used to perform clustering by function *FindClusters* and yielded 21 clusters with resolution set to 0.5. Meanwhile, the top 10 specific expressed genes of each cluster were extracted by function *FindAllMarkers* and devoted to identify the cell types. Based on the expression of canonical marker genes, we then refined the cell cluster annotations to ensure the accuracy of annotations. With the cell types identified, we performed sub‐clustering analysis on several cell types annotated primly to identify sub‐populations. To identify differentially expressed genes (DEGs) by each cluster, function *FindAllMarkers* was used with parameters ‘min.pct = 0.1’ and ‘thresh.use = 0.25’, which Wilcoxon rank sum test was inbuilt. Combination of canonical lineage markers and genes identified in differential gene expression analysis were put into sub‐population identification. Meanwhile, the top 10 dimensions resulted from PCA were used for the uniform manifold approximation and projection (UMAP) and following visualization with function *Dimplot*. For prioritize cell types, R package Augur [15] was used to calculate the Area Under the recovery Curve (AUC) scores by different comparisons.

### 2.5 SCENIC Analysis

Single‐cell regulatory network inference and clustering (SCENIC) analysis was performed on each cell type according to the pySCENIC tutorial [16]. SCENIC reconstructs gene regulatory networks (GRN) from the expression matrix of all samples. Potential TF targets based on gene co‐expression were identified, following which TF motif enrichment analysis was performed to spot the direct target known as regulons. The ranking datasets and motifs datasets were downloaded from cisTarget (v10). Thirdly, the enrichment of each regulon was measured as the AUC of the genes that define this regulon, and regulons with an average AUC score ≥ 0.1 were retained. The specific regulons that may be involved in CHB pathogenesis were presented in R package ggplot2 (v 3.4.0).

### 2.6 Differential Gene Expression, Gene Ontology and Pathway Enrichment Analysis

We extracted the averaged genes expression level of each cell type and group for differential gene expression analysis. Function *FindMarkers* was used to performed differential gene expression analysis with parameter logfc. threshold = 0.25. R package clusterProfiler (v4.6.0) [17] was used in procedure of Gene Ontology (GO) and Kyoto Encyclopedia of Genes and Genomes (KEGG) analysis, and the Bonferroni‐adjusted *p* < 0.05 was used as the cut‐off. The GO term and KEGG pathway enrichment results were presented in dot plot and heat map, and the corresponding log_2_ fold‐change values of average expression in different comparisons were visualized as heat maps by using R package ggplot2 (v3.4.0) and pheatmap (v1.0.12). To illustrate the type I interferon and fibrosis activities, function *AddModuleScore* was performed on the basic of interferon‐stimulated genes (ISGs) in Systemic Lupus Erythematosus (SLE) [18], meanwhile, fibrosis related genes were selected in published articles [19].

### 2.7 Pseudotime Analysis and Cell Communication Analysis

The R Package Monocle [20] (v.2.28.0) was used for cell trajectory and pseudotime analyses for selected cell clusters. In Brief, for cell clusters consisting of cells from four groups, the DEGs from each group in the cluster were combined and used to order genes for DDRTree analysis (function *reduceDimension*) and pseudotime ordering (function *orderCells*). Each individual cell in an analysed cluster was then assigned with a pseudotime in the end. Cell‐to‐cell communication analysis was performed using the python‐based CellphoneDB [21] package.

### 2.8 Cell Culture, RT‐qPCR and Apoptosis Level Evaluation

Human monocyte cell line THP‐1 cultured in 1640 medium (Gibco, Thermo Fisher Scientific) with 10% fetal bovine serum (Sigma) at 37°C, 5% CO_2_ atmosphere. Cells were treated with dexamethasone at concentrations of 0, 0.1 and 1 μM for 48 h, followed by analysis using real‐time quantitative PCR (RT‐qPCR) and Annexin V‐FITC flow cytometry for apoptosis detection. The positive FITC events would be recorded as the apoptotic cells.

*Thbs1* conditional knockout mice (*Thbs1* ^flox/flox^; *Lyz2*‐Cre^+/−^) were purchased from Cyagen Biosciences for bone marrow‐derived macrophages (BMDMs) isolation. Mice were euthanized by cervical dislocation and promptly immersed in 75% ethanol for 2 min. All tibiae and femora were removed, and surrounding muscle tissue was meticulously excised. Then both ends of the bones were trimmed. PBS (Gibco, Thermo Fisher Scientific) was drawn into a syringe and used to repeatedly rinse the bone marrow into a culture dish until the bones turned completely white. The bone marrow suspension was collected and filtered through a 70‐μm mesh and centrifuged at 500 *g* for 5 min. The pellet was resuspended and the lysis of red blood cells were using ACK buffer (Gibco, Thermo Fisher Scientific). Seed cells at 5 × 10^5^ cells/mL in DMEM medium (Gibco, Thermo Fisher Scientific) supplemented with 10% FBS, and 10% L929 supernatant for culture.

RNA isolation was performed using NucleoZol reagent (MACHEREY‐NAGEL) according to the manufacturer's protocol. The yield and integrity of the RNA were determined using a NanoDrop 2000c spectrophotometer. For RT‐qPCR analyses, 1 μg RNA was converted to complementary DNA using HiScript II One Step RT‐PCR Kit (Vazyme, P611) and RT‐qPCR reactions were performed on a Bio‐Rad CFX384 thermal cycler usingTaq Pro Universal SYBR qPCR Master Mix (Vazyme, Q712) and 500 nM of forward and reverse primers with the cDNA in 96‐well plates. The fold changes in the relative quantifications were normalized to the expression of 18S; the relative fold change of expression was measured using the $2^{-∆∆C_{t}}$ method where Δ*C*_t_ = *C*_t_ (detected gene) − *C*_t_ (control gene) and *C*_t_ indicated the threshold cycle number.

## 3 Result

### 3.1 Clinical Outcomes

All patients enrolled in our study had been treated with dexamethasone during the fetal period. Among them, the first one treated since the 18th week of gestation showed a normal ECG after birth. The second one treated since the 27th week still showed second‐degree AVB. Although the third one who had been treated from 31 weeks showed intermittent complete left bundle branch block, QT interval and QTc prolongation in postnatal ECG, whose whole‐exome gene report subsequently suggested a suspicious variant *KCNH2* (long QT syndrome type 2). More details were listed in Figure S1. Therefore, we excluded the third one from the patient population but used it in subsequent analyses to calibrate for immunological changes caused by dexamethasone itself.

Here, we briefly describe our clinical groupings and the corresponding actual sample sizes, including the treatment success group (CHB responsive, CHB‐r) and treatment failure group (CHB non‐responsive, CHB‐nr) within the CHB cohort, the long QT syndrome group (LQTS, representing immune baseline perturbation induced by dexamethasone) and the healthy fetal group (a total of three clinical samples were included). In the subsequent intergroup comparative analyses, samples from some groups were combined: CHB‐r and CHB‐nr were combined into a single CHB group, and the three healthy samples were combined into a single Control group. Through these different comparison strategies, we aimed to explore the immunological changes in CHB (CHB vs. LQTS), factors associated with glucocorticoid therapy failure (CHB‐nr vs. CHB‐r), and the immunological effects of dexamethasone on the fetus (LQTS vs. Control).

### 3.2 Cell Atlas of CHB Fetal Umbilical Cord Blood

**FIGURE 1:**
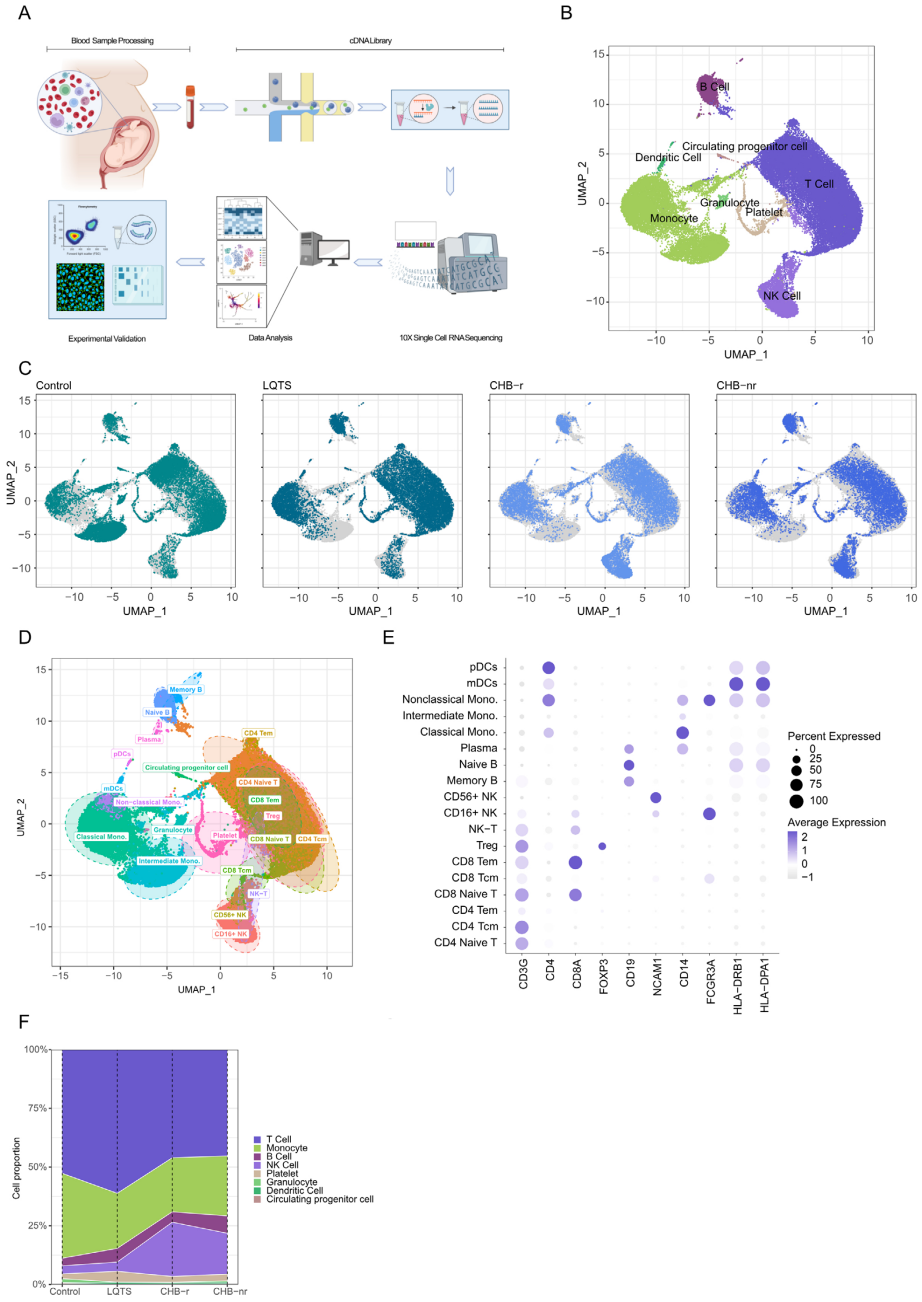
Identification of cell types in CHB. (A) Workflow for single‐cell transcriptome profiling of umbilical cord blood. (B, D) Clustering for 48,395 cells from four groups (CHB‐r, CHB‐nr, LQTS and Control) based on established lineage markers and visualized using UMAP. (C) The general patterns of UCBMC populations. (E) Dot plot showing expression levels of known lineage markers in all 48,395 cells. (F) Cell proportion diversity in four groups.

The brief process diagram had been described in Figure 1A. After filtering the data with stringent high quality, we obtained scRNA‐seq data of 15,711 cells from Control, 12,657 cells from LQTS, 10,344 cells from CHB‐r and 9683 cells from CHB‐nr. Using Uniform Manifold Approximation and Projection (UMAP), 8 cell types of umbilical cord blood mononuclear cells (UCBMCs) were identified based on the canonical marker gene (Figure 1B,C). Upon the DEGs and approved biomarkers, 8 cell types were classified into 21subsets (Figure 1D,E). T cells (*CD3G*+) were classified into naïve CD4 T cells (*RPS17*+/*IL7R*+/*ITM2A*+), naïve CD8 T cells (*CD8B*+/*LINC02446*+), effector memory CD4 T cell (CD4 Tem, *MALAT1*+/*XIST*+), central memory CD4 T cell (CD4 Tcm, *RPS17*+/*IL7R*+/ *ITM2A*+), effector memory CD8 T cell (CD8 Tem, *CD8A*+/*AC090498.1*+/*GNB2L1*+), central memory CD8 T cell (CD8 Tcm, *STMN1*+/*TUBA1B*+) and Treg (*IL32*+/*RTKN2*+/*FOXP3*+). B cells (*CD19*+) were classified into naive B cell (*IGHM*+/*IGKC*+), memory B cell (*MS4A1*+/*IL7R*+/*IL32*+) and plasma cell (*GZMB*+). NK cells (*GNLY*+/*NKG7*+) were classified into CD16+ NK (*FCGR3A*+), NK‐T (*CD3G*+/*IL32*+) and CD56+ NK (*NCAM1*+). Monocytes (*CXCL8*+/*CD14*+) were classified into classical monocyte (*S100A8*+/*S100A9*+), intermediate monocyte (*NEAT1*+/*CSF3R*+) and non‐classical monocyte (*FCGR3A*+). Dendritic cells (*CD1C*+) were classified into myeloid dendritic cells (*HLA‐DPB1*+/*HLA‐DPA1*+) and plasmacytoid DCs (pDCs, *PTGDS*+/*JCHAIN*+). At last, small amounts of granulocytes (*ITGAM*+/*DEFA3*+), platelets (*PPBP*+/*PF4*+) and circulating progenitor cells (*SPINK2*+/*AREG*+) were identified.

### 3.3 Diversity of UCBMCs in Different Condition

The general patterns of UCBMC populations were comparable across groups. The proportion of NK cells varies greatly among the groups, which was consistent with that found in a previous study for anti‐Ro/La exposed CHB neonates [9]. Another large cell population that was highly variable in innate immune cells was monocytes, of which higher percentages of classical and non‐classical monocytes were observed in LQTS, CHB‐r and CHB‐nr, which were all treated with dexamethasone compared to that in Control. There was no significant difference in the comparison of dendritic cells among the four groups. As for the adaptive immune cells, T cells accounted for the biggest proportion in this study, but they were predominantly in the naive T cell stage, the same as B cells. It is considered to be related to the fact that adaptive immunity in the neonate has not yet developed. All above, our findings indicated that innate immune mechanisms may contribute to the pathogenesis of CHB (Figure 1F).

**FIGURE 2:**
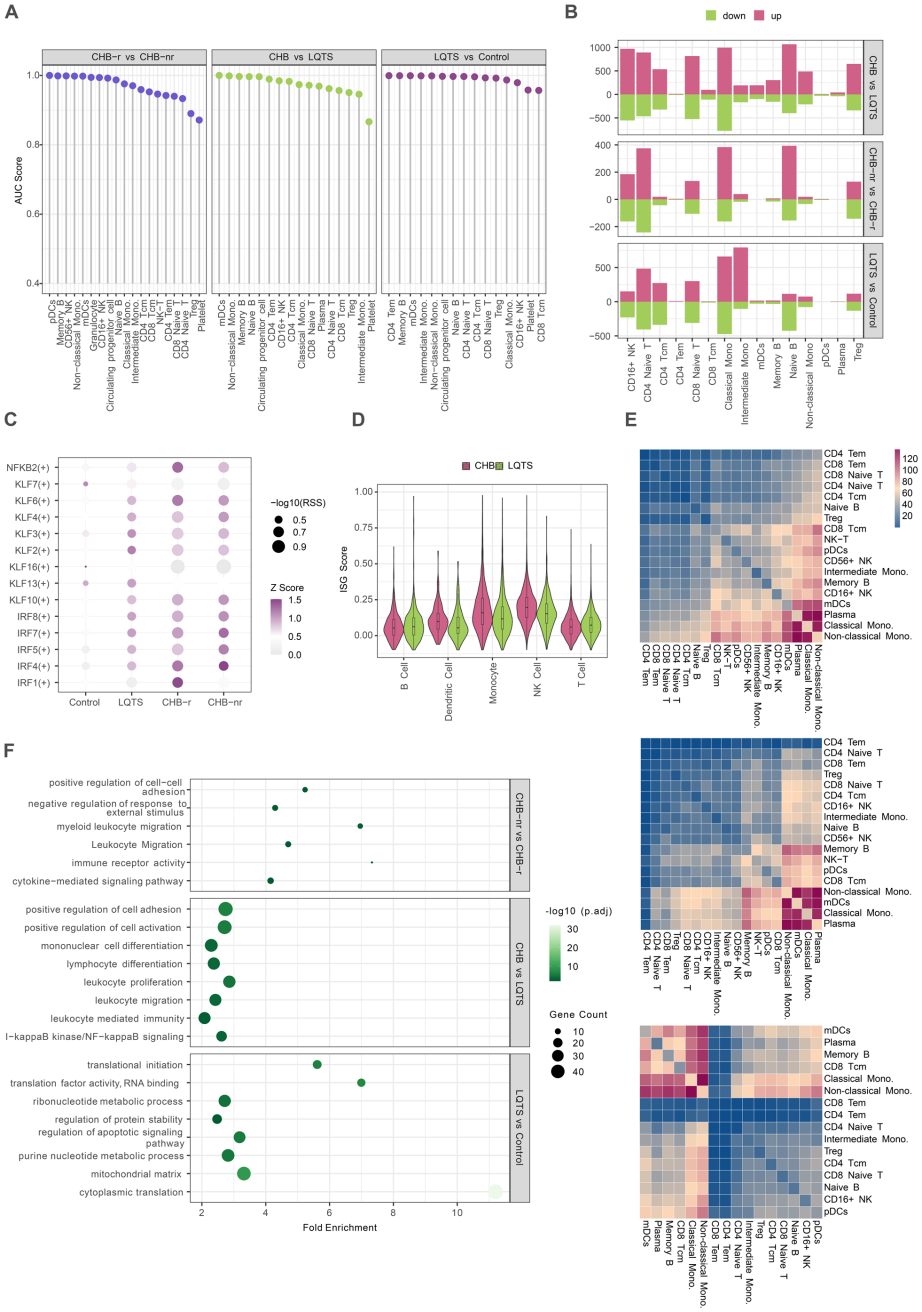
Differential expressed genes and functional analyses between conditions. (A) Cell type prioritization analysis with comparative strategies by Augur. (B) Numbers of DEGs with comparative strategies. (C) Active transcriptome regulons in different conditions using SCENIC analysis. (D) ISGs expression level in every cell type with the comparison of CHB and LQTS. (E) Cell communication differs from each condition (from top to bottom was CHB, LQTS and Control). (F) GO enrichment results displayed by comparative strategies.

To further prioritize the cell types most responsive to CHB, we applied Augur analysis on our scRNA‐seq data and found that mDCs, non‐classical monocytes, memory B cells and naïve B cells had the highest scores comparing CHB and LQTS (area under the curve [AUC] > 0.9), whereas CD4 Tem, memory B cells, mDCs, intermediate monocytes and non‐classical monocytes may be affected the most in LQTS compared to that in Control (Figure 2A). These results consistently reflect the fact that monocytes exhibit an active response under both disease perturbation and immunotherapy.

We next calculated the fraction of DEGs (p adjusted value cut‐off of 0.05) among all annotated cellular types between subgroups and their equivalent control groups (CHB‐nr vs. CHB‐r, CHB vs. LQTS, LQTS vs. Ctrl) (Figure 2B). Similarly, the DEGs in monocytes showed a consistent trend of up‐regulation between comparisons across all groups, further suggesting that the monocyte might be involved both in disease and therapeutic immune response. Although in the comparison of the CHB and LQTS groups, a higher fraction of up‐regulated DEGs was also identified in other immune cell types, including CD16+ NK, mDCs, Treg, naïve T cells, CD4 Tcm, naïve B cells and memory B cells. Although CD16+ NK and naïve B cells demonstrated more down‐regulated DEGs in LQTS compared to control groups. It suggested that CD16+ NK might be involved in the immune response of CHB disease but not in the therapeutic response to dexamethasone. CD4 Tem, CD8 Tcm, pDCs and plasma cells presented too few DEGs to analyse. NK‐T, CD56+ NK and CD8 Tem, on the other hand, were not presented because of low cell numbers.

After analysing the major cell types potentially involved in the CHB disease process, we aimed to further explore the intercellular interactions by analysing the strength of ligand–receptor interactions among various cell types using CellphoneDB. The results showed that strong interactions among classical monocytes, non‐classical monocytes, plasma cells, and mDCs were observed in the peripheral immune environment of the healthy control group (Control), the dexamethasone‐treated non‐disease group (LQTS), and the disease group (CHB) (Figure 2C). Additionally, in each sample, memory B cells and CD8+ Tcm cells also exhibited varying degrees of interaction with the aforementioned cell subsets. In the CHB group, the cell types interacting with monocyte subsets (classical monocytes, non‐classical monocytes) were more extensive, including various NK cell subsets and pDCs, all of which showed a certain degree of interaction.

Taken together with the actual responsive cell subsets identified by Augur analysis, these findings further suggest that monocytes (mainly classical and non‐classical monocytes) serve as the primary effector cells in the peripheral immune environment of CHB, whereas mDCs and CD16+ NK cells among the innate immune cells may also play a partial role.

### 3.4 Inflammation Activation Was Detected in Myeloid Cells of CHB

To investigate the functional alterations of each cell subset under different study conditions (disease status and dexamethasone treatment), we performed GO enrichment analyses (Figure 2D). In the CHB versus LQTS comparison, the upregulated DEGs in each cell subset were predominantly enriched in functions related to cell adhesion, immune cell migration and proliferation, as well as functions associated with the differentiation of specific cell types (monocytes, lymphocytes), and the NF‐κB signalling pathway, a classic pathway involved in immune regulation and inflammatory responses. These findings suggest that circulating immune cells in neonates with CHB are functionally active and exhibit significant inflammatory upregulation. In the CHB‐nr versus CHB‐r comparison, the upregulated DEGs in each cell subset were largely enriched in functions related to cell activation and inflammatory responses. In addition, the term ‘negative regulation of response to external stimulus’ suggested that the reason for treatment failure in the CHB‐nr group may be associated with negative regulation of the response to external stimuli. Overall, however, the expression levels of DEGs in this group were relatively low, and the enriched GO terms were less significant. In the LQTS versus Ctrl comparison, the DEGs in each cell subset were primarily enriched in GO terms related to mitochondrial metabolism and transcription. Additionally, the enrichment of apoptotic signalling pathways suggested that the immunological effects of dexamethasone may be associated with apoptosis.

In the GO analysis, the enrichment of IFN‐related functions or pathways did not rank prominently; however, given the well‐established role of IFN‐I in the pathogenesis of CHB, we independently scored the relative expression levels of ISGs in major immune cells and compared the scores between groups (CHB vs. LQTS) (Figure 2E). ISGs are a class of genes upregulated upon IFN stimulation and can either positively enhance or negatively regulate IFN‐mediated innate immune responses. In terms of cell types, monocytes and NK cells exhibited relatively higher ISG scores. Regarding clinical subgroups, the ISG score in CHB was higher than that in the control group (LQTS) across several major IFN‐secreting immune cell types (monocytes, NK cells and dendritic cells). Monocytes and dendritic cells are the main producers of IFN‐I, whereas NK cells primarily secrete type II interferon (IFN‐γ). Collectively, these findings suggest activation of the IFN‐I system in the peripheral circulation of children with CHB. We further performed transcription regulatory network analysis using SCENIC (Figure 2F), which similarly revealed enrichment of ISGs with positive regulatory functions on IFN, represented by *IRF1* and *IRF7*, in the CHB group. Additionally, members of the Krüppel‐like factors (KLFs) family, a group of zinc finger transcription factors closely associated with inflammatory responses, as well as NF‐κB2, were also enriched in the CHB group.

Collectively, these findings indicate a marked inflammatory upregulation in the peripheral circulation of children with CHB, with monocytes being closely involved.

### 3.5 THBS1+CD14+ Monocytes Mediate Activation of Inflammation Through Inflammasome

**FIGURE 3:**
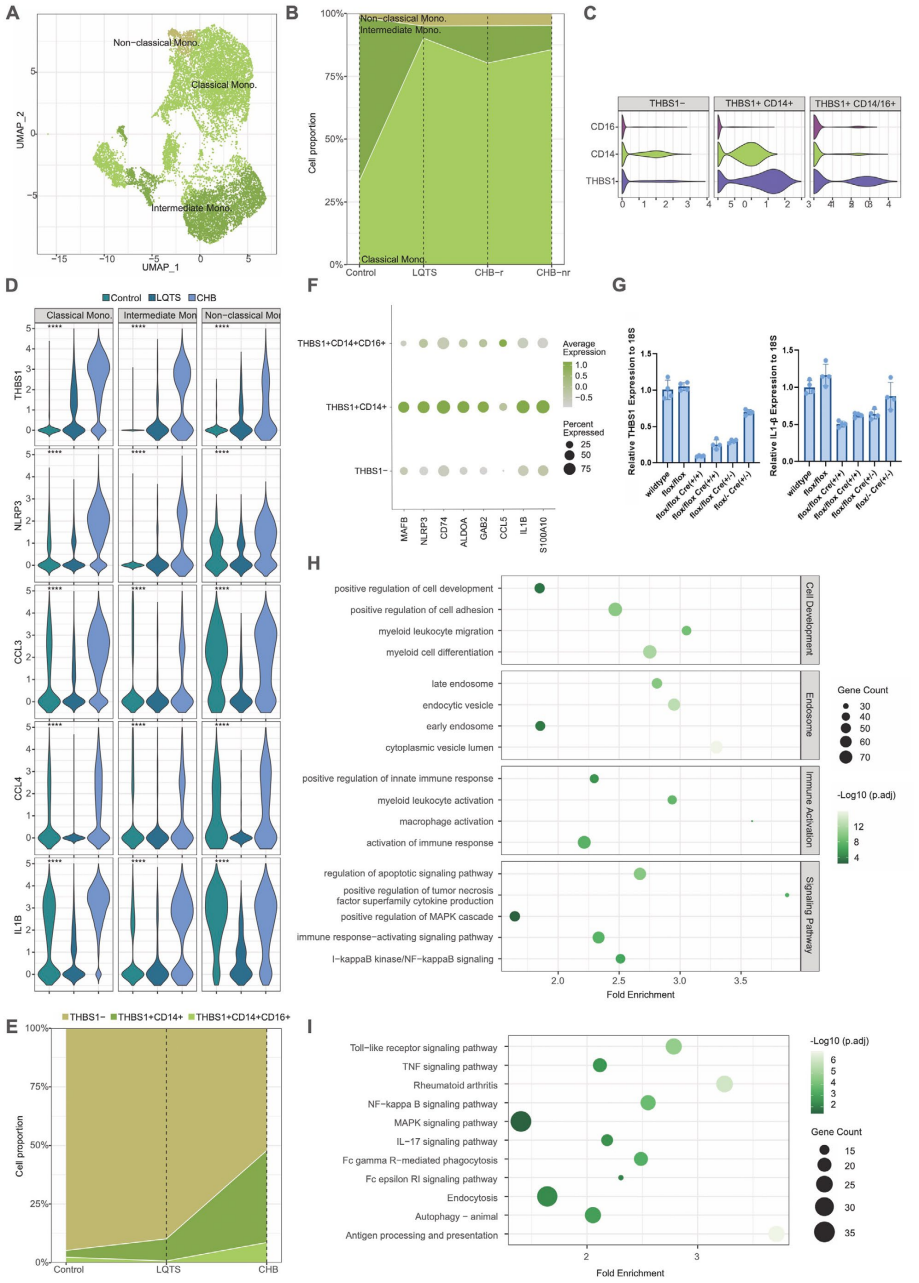
Diversity of monocytes in groups. (A) Clustering for monocytes from three conditions based on established lineage markers and visualized using UMAP. (B) Cell type proportion diversity in four groups. (C) Expression levels of DEGs in subsets (Classical Mono, Intermediate Mono, Non‐classical Mono) based on the comparison of CHB and LQTS. (D) Expression level of markers used for identifying subsets. Statistical significance was assessed using the two‐sided Wilcoxon rank‐sum test. ****p < 0.0001. The Control group is shown for descriptive purposes only and was not included in the statistical comparison. (E) Proportion diversity of monocyte subsets identified with *THBS1*, *CD14* and *CD16*. (F) Expression level of NLRP3‐inflammasome related genes. (G) The expression changes of *Thbs1* and *IL‐1β* were confirmed in BMDMs of conditional knockout mice using RT‐qPCR. (H) GO enrichment results of *THBS1*+*CD14*+ monocyte subset. (I) KEGG enrichment results of *THBS1*+*CD14*+ monocyte subset.

We performed a second‐round clustering on monocytes and firstly identified an additional small cluster of monocytes based on the expression of classic biomarkers (*CD14*, *CD16*). Among the clinical groups, donors from the LQTS, CHB‐r and CHB‐nr groups showed an increased proportion of classical monocytes but a reduced fraction of intermediate monocytes (Figure 3A,B). After differential gene analysis, we found the monocytes from CHB had high expression levels of *THBS1*, *NLRP3*, *IL‐1β*, *CXCL2*, *CCL3* and *CCL4*, all of which were importantly associated with inflammatory responses (Figure 3D). We then identified *THBS1* as a new cell‐clustering marker for monocytes, which subsequently were classified into *THBS1*− monocytes, *THBS1*+*CD14*+ monocytes and a small amount of *THBS1*+*CD14*+*CD16*+ monocytes (Figure 3C). The proportion of *THBS1*+*CD14*+ monocytes was increased significantly in both CHB‐r and CHB‐nr patients (Figure 3E). Next, we systematically identified the DEGs between *THBS1*+*CD14*+ monocytes and all the other monocytes. Apart from *CCL5*, *CD74* and other highly expressed genes associated with inflammatory responses, *NLRP3* and *IL‐1β* were again significantly upregulated in *THBS1*+*CD14*+ monocytes (Figure 3F). We have employed RT‐qPCR to examine the expression changes of *Thbs1* and *IL‐1β* in BMDMs from conditional knockout mice. Our results demonstrated that BMDMs from *Lyz*‐Cre;*Thbs1* ^flox/flox^ mice exhibited reduced *IL‐1β* levels, providing preliminary evidence supporting an association between *Thbs1* and *IL‐1β*—a key downstream effector of the NLRP3 inflammasome pathway—in murine macrophages (Figure 3G).

Functional enrichment tests indicated that *THBS1*+*CD14*+ monocytes were enriched with upregulated genes related to the inflammatory response and immune activation, including NF‐kappa B signalling pathway, a vital step in the activation of NLRP3 inflammasome pathway (Figure 3H,I).

**FIGURE 4:**
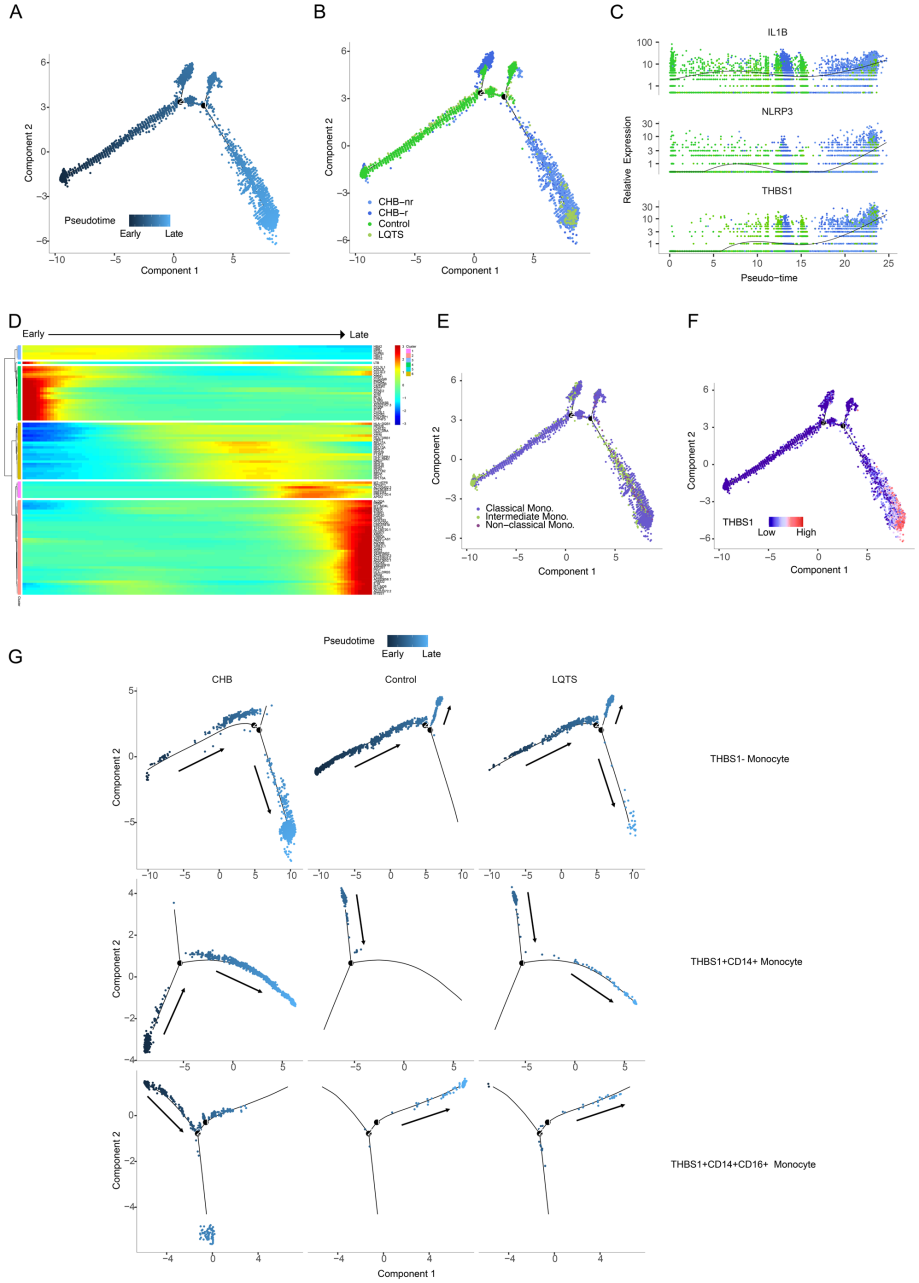
Cell trajectory analyses among monocytes. (A) Trajectory of monocytes along pseudotime in a two‐dimensional space. Each point corresponds to a single cell. (B) Trajectory of monocytes along condition in a two‐dimensional space. (C) Expression changes of *THBS1*, *IL‐1β* and *NLRP3* displayed along pseudotime. (D) Heatmap showing the dynamic changes of gene expression along pseudotime. The DEGs were clustered hierarchically into six groups. (E) Trajectory of monocytes along monocyte subsets (Classical Mono, Intermediate Mono, Non‐classical Mono) in a two‐dimensional space. (F) Expression of *THBS1* genes in monocyte trajectory. (G) Trajectory of monocyte subset (*THBS1*− Mono, *THBS1*+*CD14*+ Mono, *THBS1*+*CD14*+*CD16*+ Mono) along condition in a two‐dimensional space.

To explore the dynamic changes and differentiation trajectories of monocyte cell states under stimulation (including disease and drug treatment), we performed pseudotime analysis on monocytes, showing the differentiation and developmental trajectory of monocytes (Figure 4A). Corresponding to the clinical subgroups (Figure 4B), cells from the healthy control group (Control) were mainly located at the early differentiation stage, whereas clinical subgroups exposed to dexamethasone or disease stimuli (LQTS, CHB‐nr and CHB‐r) were predominantly at the late differentiation stage. Pseudotime analysis of these three genes across clinical subgroups (Figure 4C) revealed nearly identical expression trends, with markedly high expression in the disease groups, suggesting that monocyte subsets at the late differentiation stage in children with CHB highly express inflammation‐related genes. Based on the pseudotime expression dynamics of genes, we identified six distinct gene expression modules (Clusters 1–6, Figure 4D). Genes in Modules 3, 4 and 5 were downregulated along pseudotime, whereas Modules 6 and 1 contained genes with the highest expression during the mid‐to‐late transition period, and Module 2 represented genes upregulated at the terminal stage. Within Module 2, we again noted high expression of *THBS1* as well as *IL‐1β* and *NLRP3*, two key genes associated with the inflammasome.

In terms of cell subsets (Figure 4E), each of the conventionally classified monocyte subsets (classical, intermediate and non‐classical monocytes) exhibited relatively complete differentiation, with non‐classical monocytes mainly distributed in the mid‐to‐late differentiation stage, whereas classical monocytes remained the most abundant subset at the late differentiation stage. With respect to the pseudotime distribution of *THBS1* gene expression (Figure 4F), *THBS1* was highly expressed at the late stage of monocyte differentiation, corresponding to the stage of functional maturation. In summary, both disease and drug stimuli induced monocyte differentiation, with monocytes at the late differentiation stage showing high expression of *THBS1*, predominantly in classical monocytes (*CD14*+). We also visualized the differentiation trajectories of the newly defined monocyte subsets (*THBS1*− monocyte, *THBS1*+*CD14*+ monocyte and *THBS1*+*CD14*+*CD16*+ monocyte) by subgroup (Figure 4G), which again indicated that clinical subgroups with disease status or dexamethasone intervention (CHB and LQTS) exhibited more mature differentiation.

In summary, pseudotime analysis indicates that monocytes from children with CHB exhibit a more mature differentiation state and display a pro‐inflammatory phenotype.

### 3.6 Dexamethasone Facilitated Apoptosis of Immune Cells

**FIGURE 5:**
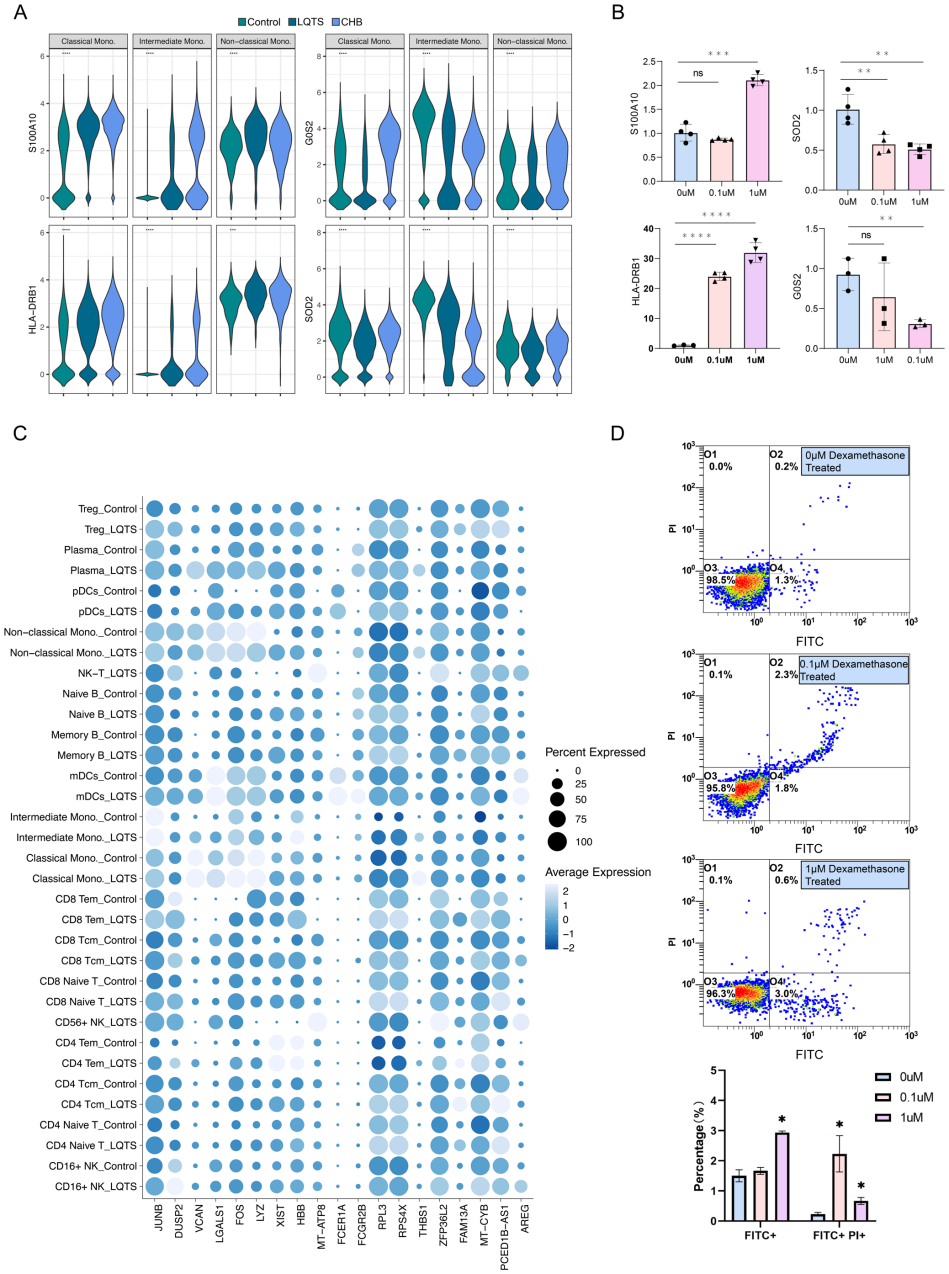
Comparison of LQTS and Control revealed the activation of apoptosis. (A) Expression levels of DEGs in subsets (Classical Mono, Intermediate Mono, Non‐classical Mono) based on the comparison of LQTS and Control. Statistical significance was assessed using the two‐sided Wilcoxon rank‐sum test. ***p < 0.001, ****p < 0.0001. The CHB group is shown for descriptive purposes only and was not included in the statistical comparison. (B) The expression changes of DEGs were confirmed in THP‐1 with dexamethasone treatment using RT‐qPCR. Statistical significance was assessed using one‐way analysis of variance (ANOVA) followed by Dunnett's post hoc test, comparing each treatment group to the control group (0 μM), ns, not significant, **p < 0.01, ***p < 0.001, ****p < 0.0001. (C) Expression levels of top 2 DEGs in each cell subset based on the comparison of LQTS and Control. (D) Flow cytometry revealed the apoptosis level changes of THP‐1 with dexamethasone treatment using Annexin‐V/PI. Statistical significance was assessed using one‐way analysis of variance (ANOVA) followed by Dunnett's post hoc test, comparing each treatment group to the control group (0 μM), *p < 0.1.

Monocytes are a key immune cell type in this study. Previous analyses have shown their involvement in both immune inflammatory responses and apoptosis; we therefore hypothesized that monocytes may serve as important responder cells for the immunological effects of dexamethasone. Here, we selected four representative DEGs in monocytes between the LQTS and Control groups for individual presentation (Figure 5A). Both *S100A10* and *HLA‐DRB1*, which are associated with monocyte/macrophage function, showed increased expression in the LQTS group compared with the control group, suggesting that dexamethasone stimulates immune responses in monocytes. In contrast, *SOD2* and *GOS2*, both related to apoptosis, exhibited overall downregulation in the LQTS group relative to the control group, indicating that the immunological effects of dexamethasone may include an impact on apoptosis. RT‐qPCR revealed upregulation of *S100A10* and *HLA‐DRB1* and downregulation of *SOD2* and *GOS2* in THP‐1 cells following dexamethasone treatment (Figure 5B), preliminarily validating the aforementioned bioinformatics findings. Based on the comparison between LQTS and Control, we tried to figure out the effect on dexamethasone treatment. Differential gene expression analysis indicated that, compared with the control group (Control), the upregulated DEGs in most cell subsets from LQTS samples were primarily associated with immune regulation (*JUNB*, *FOS* and *LGALS1*), regulation/promotion of apoptosis (*VCAN*, *DUSP2* and *AREG*), protein synthesis (*RPL3*, *RPS4X*) and mitochondrial function (*MT‐ATP8*, *MTCYB*), suggesting that dexamethasone may stimulate cellular activity, immune responses, and apoptosis in immune cells (Figure 5C), which is consistent with the results of the GO enrichment analysis described above. Flow cytometry analysis also demonstrated apoptosis in THP‐1 cells after dexamethasone intervention (Figure 5D). It revealed the administration of dexamethasone would induce monocyte apoptosis to reduce associated immunological attacks, which provided potential evidences for the application of dexamethasone in CHB management.

### 3.7 The Alternations of Lymphoid Cells Contribute to Myocardial Fibrosis

**FIGURE 6:**
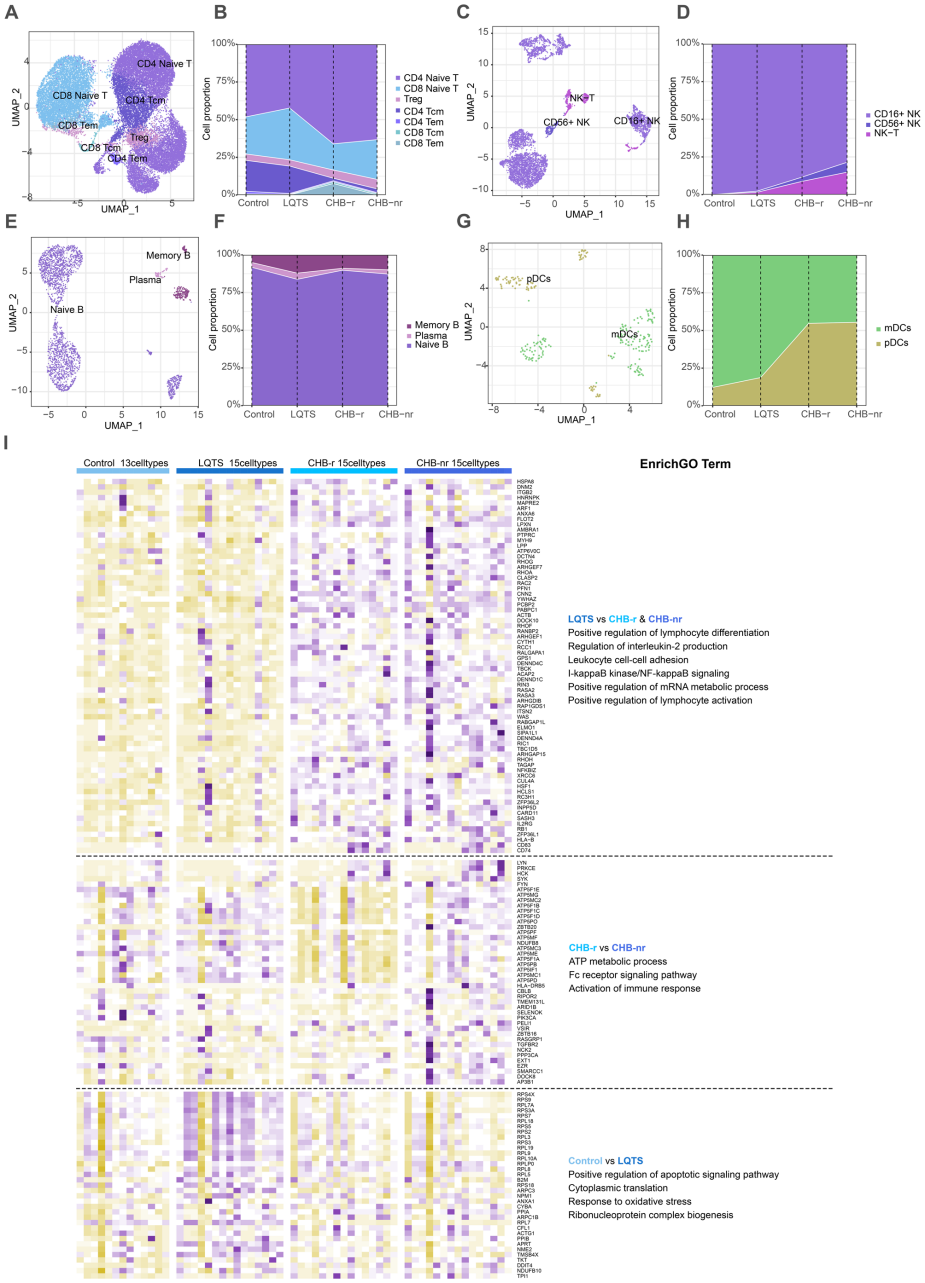
Identification and functional analyses of lymphoid cells and dendritic cells between conditions. (A–H) Integrated UMAP graph and fraction of lymphoid cells and dendritic cells in the sequenced cells among different groups. (I) Heatmap showing the dynamic changes of gene expression along condition in 15 cell subsets. The DEGs were put into GO enrichment analysis and representative enriched pathways of each group were shown.

A based on the aforementioned findings, THBS1‐expressing CD14+ monocytes were identified as the predominant cell population contributing to the pathological progression of CHB. To further elucidate the cellular functions of other immune cell types, we performed comprehensive subtype analyses across the major lymphoid and myeloid populations. Regarding T cell heterogeneity, unsupervised clustering revealed seven distinct subpopulations, including CD4+ naïve T cells, CD8+ naïve T cells, Tregs, CD4+ central memory T cells (CD4+ Tcm), CD4+ effector memory T cells (CD4+ Tem), CD8+ central memory T cells (CD8+ Tcm) and CD8+ effector memory T cells (CD8+ Tem) (Figure 6A). Notably, the proportion of CD4+ Tcm was significantly reduced in CHB foetuses, whereas CD4+ naïve T cells were markedly enriched in CHB patients (Figure 6B). With respect to natural killer (NK) cell populations, three distinct subsets were identified, comprising CD16+ NK cells, CD56+ NK cells and NK‐T cells (Figure 6C), with the proportion of NK‐T cells notably elevated in CHB patients (Figure 6D). Further analysis of B cell composition revealed three distinct clusters, memory B cells, plasma cells and naïve B cells (Figure 6E), with no significant alterations in the relative proportions of B cell subpopulations observed among CHB cases (Figure 6F). Dendritic cells (DCs) were classified into two major subsets, namely myeloid DCs (mDCs) and plasmacytoid DCs (pDCs) (Figure 6G), with pDCs demonstrating a statistically significant increase in CHB patients (Figure 6H). To systematically characterize the functional states of lymphoid cells across the study cohort, GO enrichment analyses were conducted for each identified cellular subtype across the four patient groups. The results demonstrated that lymphoid cells in CHB patients exhibited pronounced activation of lymphocyte differentiation pathways and augmented cytokine production, processes that are mechanistically implicated in myocardial fibrosis underlying conductive disorders. In contrast, treatment‐responsive foetuses displayed attenuated immune responses accompanied by downregulation of ATP metabolic processes. Meanwhile, PBMCs derived from LQTS patients exhibited heightened apoptotic signalling and elevated oxidative stress (Figure 6I). Collectively, although lymphoid cells were not identified as the primary determinants of fetal CHB onset, these findings underscore their regulatory roles in modulating immune activity and cytokine production within the broader pathophysiological context of fetal conductive disorders.

## 4 Discussion

Previous studies on the pathogenesis have focused on local immune damage and inflammatory responses triggered by immune complexes formed by maternal autoantibodies in the fetal cardiac conduction system. However, this mechanism does not fully account for all cases of disease onset or non‐onset, as an immunological response to underlying pathogenic factors should also exist even in cases with negative maternal autoantibodies. In this study, we expanded the conceptual framework of iCHB to encompass systemic immune activation and inflammatory responses in the fetus.

In this study, we characterized the major immune cell populations in umbilical cord blood from neonates with CHB and found that CHB is associated with compositional alterations in circulating immune cells, predominantly within the innate immune compartment, whereas adaptive immune cells (T and B lymphocytes), although accounting for a larger proportion overall, mainly existed in a naïve state. Activation of the innate immune system in CHB has indeed begun to be recognized in recent years, with monocytes/macrophages receiving sustained attention. A recent study reported that Sialic acid‐binding immunoglobulin‐like lectin‐1 (SIGLEC‐1, a marker of monocyte activation inducible by IFN‐I)‐positive macrophages may contribute to the inflammatory process in the fetal heart in CHB by activating IFN‐I and proposed that SIGLEC‐1+ macrophages may amplify the disease effects in CHB through recruitment of additional monocytes [10]. Another study demonstrated activation of CD14+SIGLEC‐1+ monocytes, activation of the IFN‐I system, and elevated plasma IFNα levels in umbilical cord blood from neonates exposed to anti‐Ro/La antibodies, suggesting that circulating monocytes are also activated in foetuses at high risk for CHB [22]. However, the role of circulating monocytes in the pathogenesis of CHB requires further evaluation. Our study revealed that monocytes represent an important cell type within UCBMCs. Compared with the control group, CHB patients showed upregulation of differentially expressed genes in both classical and non‐classical monocyte subsets, which exhibited a high response priority and were the cell type with the strongest interactions with other immune cells. GO enrichment analysis identified monocyte differentiation as a significantly enriched term. Together, these findings indicate that monocytes represent a key immune cell type within the broadly activated innate immune system in CHB.

In addition to identifying key immune cell types in CHB, we also found that a pronounced inflammatory response is indeed present in CHB and appears to be closely linked to the monocyte/macrophage population. First, we confirmed the potential important role of the classical IFN‐I system in the pathogenesis of CHB. Transcription factor enrichment analysis revealed that, compared with the control group, interferon regulatory factors (IRFs) with positive regulatory functions on IFN, such as *IRF1* and *IRF7* [23, 24], were enriched in neonates with CHB. Monocytes are the primary producers of IFN‐I, and in this study, monocytes from CHB patients showed higher ISG scores than those from the control group. Moreover, when we utilized scRNA‐seq to explore the immune inflammatory environment in CHB, we uncovered a broader inflammatory response network. GO enrichment analysis indicated the enrichment of the NF‐κB signalling pathway, which is closely associated with immune regulation and inflammatory responses, in neonates with CHB. NF‐κB mediates the induction of various pro‐inflammatory genes in innate immune cells, serves as a key transcription factor for M1 macrophages, and is essential for inducing a broad array of inflammatory genes (e.g., *TNF‐α*, *IL‐1β*) [25]. Members of the KLF family, which are closely related to inflammatory responses (e.g., *KLF6*, *KLF4*), as well as the transcription factor NF‐κB2, were also enriched in neonates with CHB. It has been reported that *KLF6* participates in regulating macrophage polarization and triggering pro‐inflammatory responses [26]. *KLF4* acts as a mediator of pro‐inflammatory signalling pathways in macrophages and is closely associated with numerous inflammatory factors. In macrophages, cytokines such as IFN‐γ can significantly induce *KLF4* expression [27]. Furthermore, *KLF4* is a key regulator in the transcriptional network controlling monocyte differentiation. In vitro, *KLF4* deficiency impairs monocyte differentiation; conversely, *KLF4* activates the monocyte‐specific CD14 promoter.

Thus, we hypothesized that monocytes may drive the systemic inflammatory response in CHB. We therefore proceeded to conduct further analyses on monocytes. Monocytes are composed of subsets with distinct phenotypes and functions, and their heterogeneity enhances our understanding of inflammatory pathogenesis, with increases in specific subset proportions potentially serving as disease biomarkers. Functionally, classical monocytes (*CD14*++*CD16*–) are responsible for enhanced phagocytosis, exhibit anti‐apoptotic properties, and possess pro‐inflammatory capacity [28]; they have also been shown to differentiate into macrophages [29]. In our study, we observed a marked increase in the classical monocyte subset in CHB compared with controls. GO and KEGG enrichment analyses revealed enrichment of multiple signalling pathways closely associated with pro‐inflammatory responses in monocytes in CHB including the NF‐κB, MAPK, TNF and Toll‐like receptor signalling pathways. Pseudotime analysis further indicated that, compared with controls, monocytes (predominantly classical monocytes) in CHB exhibited a more mature differentiation state and highly expressed inflammation‐related genes at the late differentiation stage.

During differential gene expression (DGE) analysis of monocytes, we observed that inflammation‐related genes such as *THBS1*, *NLRP3*, *IL‐1β*, and the monocyte chemokines *CCL3* and *CCL4* were markedly upregulated in CHB. Thrombospondin‐1 (THBS1 or TSP1) is a multifunctional glycoprotein released from platelets, macrophages and adipocytes [30], and is involved in a wide range of physiological and pathological processes, including cell adhesion, inhibition of angiogenesis and endothelial cell proliferation [31], and regulation of inflammation [32, 33]; it can also activate TGF‐β [34]. In a mouse model of abdominal aortic aneurysm, THBS1 was shown to play a critical role in regulating macrophage adhesion, migration and recruitment in inflammatory responses [35]. Another study found that in a mouse model of hypoxia‐induced pulmonary hypertension, circulating monocytes recruited to the lungs expressed THBS1, which activated TGF‐β [36]. We therefore hypothesized that THBS1 may be associated with the pro‐inflammatory phenotype of monocytes. Based on this molecular signature, combined with canonical monocyte marker genes (*CD14*, *CD16*), we reclassified monocytes into three subsets: *THBS1*− monocytes, *THBS1*+*CD14*+ monocytes, and *THBS1*+*CD14*+*CD16*+ monocytes. DGE analysis following this reclassification showed that the *THBS1*+*CD14*+ cell subset exhibited upregulation of multiple genes associated with inflammatory responses, including *NLRP3* and *IL‐1β*. NOD‐like receptor thermal protein domain associated protein 3 (NLRP3) is a key component of the NLRP3 inflammasome, and IL‐1β is a pro‐inflammatory cytokine produced downstream. The parallel upregulation of *THBS1*, *NLRP3* and *IL‐1β* in circulating monocytes in the disease group suggests that THBS1 may serve not only as a novel biomarker for monocytes but also as a pathogenic factor potentially involved in multiple inflammatory pathways. We thus hypothesized that THBS1 may also serve as a molecular signature of macrophages in the local cardiac tissue during CHB. RT‐qPCR results showed reduced *IL‐1β* levels in bone marrow‐derived macrophages from *Lyz*‐Cre;*Thbs1* ^flox/flox^ mice, providing preliminary evidence for an association between Thbs1 and the NLRP3 inflammasome pathway in murine macrophages.

The NLRP3 inflammasome is currently the most extensively studied inflammasome and is primarily expressed in neutrophils, monocytes, macrophages and conventional dendritic cells [37]. It can be activated by a wide range of stimuli, including reactive oxygen species stress, microbial toxins and atherosclerosis‐related cholesterol crystals [38, 39]. The activation of the NLRP3 inflammasome is generally considered to require two signals: priming and activation [40]. The priming signal can be delivered through Toll‐like receptors or cytokine receptors (e.g., TNFR1) to activate the NF‐κB signalling pathway, which in turn promotes the transcription and translation of NLRP3 and the pro‐inflammatory cytokine pro‐IL1β, and may also induce post‐translational modifications of NLRP3. The activation signal, mediated by the aforementioned stimuli, triggers inflammasome assembly, ultimately leading to the production of the pro‐inflammatory cytokines IL‐1β and IL‐18 [41]. These potent pro‐inflammatory cytokines initiate inflammatory cascades, resulting in the recruitment of innate immune cells and also shaping the subsequent adaptive immune response. Revisiting the previous hypothesis [8], during the cardiac inflammatory process in CHB, cardiomyocyte apoptosis leads to the exposure of intracellular Ro60 autoantigens, which form immune complexes with maternal autoantibodies. These complexes are phagocytosed by infiltrating macrophages via FcγR‐mediated phagocytosis and subsequently engage endosomal TLRs (TLR7/8). In vitro experimental results [42] have confirmed that TLR7/8 activation stimulates multiple signalling pathways, including the NF‐κB pathway, leading to the secretion of large amounts of pro‐inflammatory (e.g., IFN, TNF‐α) and pro‐fibrotic (e.g., TGF‐β) cytokines. Integrating these findings with our new single‐cell transcriptomic analysis, we speculate that the initial pathogenic stimuli in CHB—such as maternal anti‐Ro/La antibodies—may induce a certain degree of inflammation in the fetus. This, in turn, may activate the NLRP3 inflammasome via NF‐κB signalling (dependent on TLR signalling) or directly trigger inflammasome activation, leading to the secretion of pro‐inflammatory cytokines such as IFN, IL‐1β and IL‐18 by immune cells (predominantly monocytes/macrophages). These events initiate downstream inflammatory cascades, apoptosis and subsequent fibrotic processes, collectively contributing to irreversible pathological changes in the cardiac conduction system. In the peripheral circulation of foetuses with CHB, this excessive inflammatory response may be driven primarily by *THBS1*+*CD14*+ monocytes; meanwhile, within the pathological process affecting the cardiac conduction system, THBS1 may also represent a molecular signature of local macrophages. The identification of novel disease pathways and biomarkers may offer new therapeutic strategies or targets for CHB.

Dexamethasone exerts potent anti‐inflammatory effects primarily by suppressing various downstream inflammatory pathways (e.g., NF‐κB) through the ubiquitously expressed glucocorticoid receptor (GR), thereby reducing immune cell activity and inflammatory factor production [43]. Studies have reported that immunomodulatory therapy in mothers positive for anti‐Ro/La autoantibodies can attenuate the fetal IFN response [22]. Additionally, glucocorticoid therapy has been shown to be effective in treating NLRP3‐associated autoinflammatory diseases [44]. In CHB, dexamethasone is an important yet controversial therapeutic agent; understanding its specific mechanism of action may help optimize treatment strategies. In this study, we performed comparative analyses between samples from patients diagnosed with long QT syndrome (LQTS) who received dexamethasone treatment and healthy control samples (Control). Although this analysis, which excluded the disease context of CHB, may yield partial findings, it reduced the complexity of the analytical variables and at least allowed us to preliminarily explore the immunological effects of dexamethasone on circulating monocytes in the fetal periphery, providing a reference for the judicious use of glucocorticoids during the fetal period. Our bioinformatics analysis suggested that in individuals without an underlying immunological aetiology, the immunological effects of dexamethasone are associated with the regulation of apoptosis, with monocytes serving as important responder cells. Flow cytometry results further confirmed that dexamethasone induced apoptosis in THP‐1 cells. Previous in vitro studies have shown that dexamethasone induces reactive oxygen species production and mitochondrial‐dependent apoptosis in pro‐inflammatory macrophages via KLF9 [45]. We therefore hypothesize that in CHB, the therapeutic effect of dexamethasone may involve not only the reduction of inflammatory factor production and release but also the promotion of apoptosis in immune cells, particularly pro‐inflammatory monocytes/macrophages. In addition, GO terms related to metabolism, protein translation and ribonucleoprotein complex biogenesis were significantly enriched, indicating that dexamethasone treatment indeed impacts cellular homeostasis. In summary, despite its widespread clinical use, dexamethasone administration remains a double‐edged sword due to its disruption of homeostasis and potential adverse effects. Furthermore, one case in our cohort showed no response to dexamethasone treatment, and our analysis did not reveal substantial clues regarding this non‐response. Upon reviewing the clinical course, we noted that dexamethasone was initiated at 27 weeks of gestation in this case, suggesting that treatment may have been initiated beyond the therapeutic window, after irreversible fibrosis of the cardiac conduction tissue had already occurred. Future studies incorporating a larger sample size may uncover additional potential mechanisms underlying dexamethasone non‐response.

The present study has several limitations. First, our sample size was small with insufficient biological replicates, which limits the generalizability of our conclusions. This is primarily attributable to the low incidence of CHB. Future studies incorporating a larger number of CHB clinical samples may enable more rigorously controlled comparisons. Second, scRNA‐seq captures the single‐cell transcriptome at a specific time point; our findings reflect the outcomes of CHB progression and treatment, but cannot fully represent the humoral immune status at the initial stage of the disease. We used LQTS as a treatment control to partially account for the effects of glucocorticoid therapy, but this may have diluted the specific disease‐related effects. Nonetheless, the impact of the disease itself remains evident, and our results at least demonstrate differences from the control group, which provide insights into pathogenesis. Future inclusion of cases from different disease stages and application of additional analytical approaches (e.g., temporal transcriptomics) may yield deeper insights into CHB pathogenesis. Moreover, exploration of the specific mechanisms underlying the disease remains at a preliminary stage. Although our bioinformatics analyses revealed disease‐associated clues, further experimental studies are needed to elucidate the precise mechanistic roles of these observations. Finally, our analysis did not thoroughly investigate other circulating immune cells in CHB. For instance, NK cells may represent another innate immune cell population significantly involved in disease pathogenesis and warrant focused exploration in future studies.

## 5 Conclusion

Our study provides the first systematic depiction of the circulatory immune cell landscape in iCHB patients with maternal antibody negativity following dexamethasone therapy, filling the gap in the characterization of peripheral circulation immunity. This study revealed that the peripheral circulation in iCHB is characterized by innate immune cell activation, predominantly involving monocyte responses, accompanied by systemic inflammatory activation, including IFN signalling. THBS1 is a novel biomarker we identified in monocytes. In the peripheral circulation of CHB, *THBS1*+*CD14*+ monocytes exhibit a pronounced pro‐inflammatory phenotype associated with NLRP3 inflammasome pathway‐mediated inflammatory responses, and may also be critical in mediating interactions with local immune inflammatory responses in the heart.

## Author Contributions

**Chuan Wang:** investigation, formal analysis. **Yifei Li:** conceptualization, investigation, funding acquisition, validation, methodology, visualization, writing – review and editing, project administration, supervision, data curation, resources. **Sha Lin:** conceptualization, investigation, writing – original draft, methodology, visualization, validation, formal analysis, data curation, software. **Kaiyu Zhou:** conceptualization, investigation, methodology, validation, visualization, formal analysis, supervision, project administration, writing – review and editing. **Jie Tang:** conceptualization, investigation, validation, visualization, formal analysis, data curation, software, writing – original draft. **Haiyan Yu:** conceptualization, investigation, data curation, supervision, formal analysis, project administration, methodology, writing – review and editing, resources. **Yimin Hua:** investigation, visualization, formal analysis, project administration.

## Funding

This work was supported by grants from the National Natural Science Foundation of China (82270249, 82470249). The funding did not participate in the design of the study and collection, analysis, and interpretation of data and in writing the manuscript.

## Consent

Informed consent was obtained from the donors and their guardians.

## Conflicts of Interest

The authors declare no conflicts of interest.

## Supporting information


**Figure S1:** Clinical detail information of patients recruited in the study. In this study, umbilical cord blood samples were collected from three neonates who were diagnosed with CHB during the fetal period and treated with dexamethasone. All three patients were diagnosed with second‐degree type II atrioventricular block by fetal echocardiography during the fetal period, without pathogenic structural cardiac abnormalities, and dexamethasone treatment was initiated after referral to our outpatient clinic (either immediately or within 2 weeks after diagnosis). Maternal autoantibody tests during pregnancy were all negative, and there was no history of common viral or bacterial infections; detailed clinical information is provided in the table. The clinical outcomes of the three patients differed: Patient 1 had a normal postnatal electrocardiogram (ECG), indicating successful treatment; Patient 2 still showed second‐degree type II atrioventricular block on repeated postnatal ECGs, suggesting treatment failure; and Patient 3 was revised to a diagnosis of long QT syndrome (LQTS) based on postnatal ECG and whole‐exome sequencing, representing immune baseline perturbation induced by dexamethasone. Healthy control umbilical cord blood samples were obtained from three healthy full‐term newborns at the First Affiliated Hospital of Jinan University, and details are omitted here.

## Data Availability

The data that support the findings of this study are available from the corresponding author upon reasonable request.
