# Supplementary figures and images for: Single Cell RNA Sequencing Reveals THBS1+CD14+ Monocyte Modulates Inflammatory Activation via NRLP3‐Inflammasome in Congenital Heart Block
Source: J Cell Mol Med. 2026 Jul 21;30(14):e71270. doi: 10.1111/jcmm.71270 (PMC13389435; doi:10.1111/jcmm.71270)

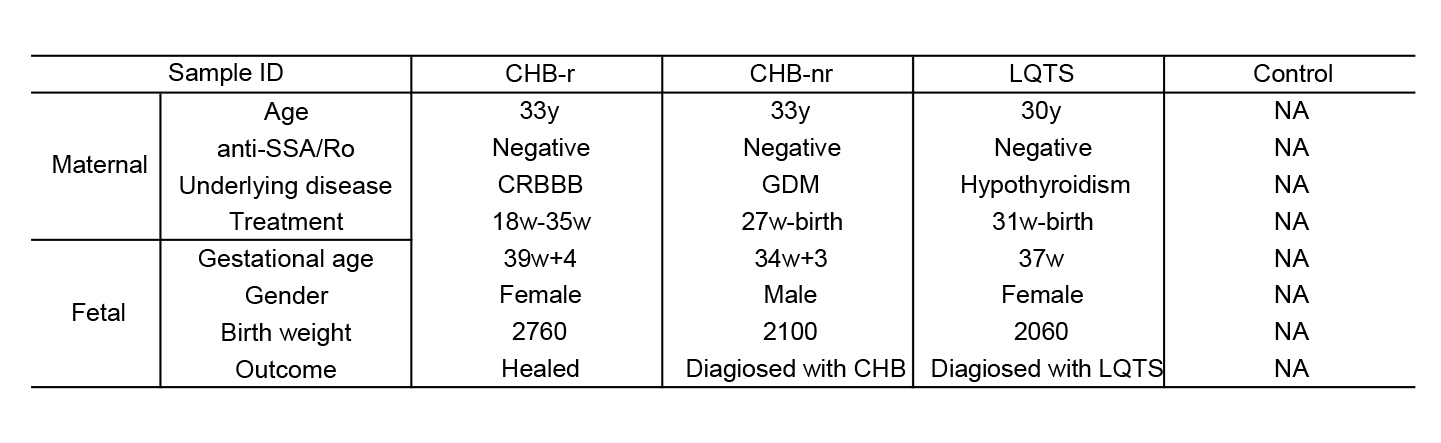

Supplement: Supplementary file 1 — Figure S1: Clinical detail information of patients recruited in the study. In this study, umbilical cord blood samples were collected from three neonates who were diagnosed with CHB during the fetal period and treated with dexamethasone. All three patients were diagnosed with second‐degree type II atrioventricular block by fetal echocardiography during the fetal period, without pathogenic structural cardiac abnormalities, and dexamethasone treatment was initiated after referral to our outpatient clinic (either immediately or within 2 weeks after diagnosis). Maternal autoantibody tests during pregnancy were all negative, and there was no history of common viral or bacterial infections; detailed clinical information is provided in the table. The clinical outcomes of the three patients differed: Patient 1 had a normal postnatal electrocardiogram (ECG), indicating successful treatment; Patient 2 still showed second‐degree type II atrioventricular block on repeated postnatal ECGs, suggesting treatment failure; and Patient 3 was revised to a diagnosis of long QT syndrome (LQTS) based on postnatal ECG and whole‐exome sequencing, representing immune baseline perturbation induced by dexamethasone. Healthy control umbilical cord blood samples were obtained from three healthy full‐term newborns at the First Affiliated Hospital of Jinan University, and details are omitted here. [file JCMM-30-e71270-s001.jpg]
